# Direct evidence for heme-assisted solid-state electronic conduction in multi-heme *c*-type cytochromes†
†Electronic supplementary information (ESI) available. See DOI: 10.1039/c8sc01716f


**DOI:** 10.1039/c8sc01716f

**Published:** 2018-07-27

**Authors:** Kavita Garg, Mihir Ghosh, Tamar Eliash, Jessica H. van Wonderen, Julea N. Butt, Liang Shi, Xiuyun Jiang, Futera Zdenek, Jochen Blumberger, Israel Pecht, Mordechai Sheves, David Cahen

**Affiliations:** a Department of Materials and Interfaces , Weizmann Institute of Science , Rehovot , Israel . Email: david.cahen@weizmann.ac.il; b Department of Organic Chemistry , Weizmann Institute of Science , Rehovot , Israel . Email: mudi.sheves@weizmann.ac.il; c School of Chemistry , School of Biological Sciences , University of East Anglia , Norwich Research Park , Norwich , NR4 7TJ , UK; d Department of Biological Sciences and Technology , School of Environmental Sciences , China University of Geosciences , Wuhan , China 430074; e Department of Physics and Astronomy and Thomas Young Centre , University College London , Gower Street , London WC1E 6BT , UK; f Department of Immunology , Weizmann Institute of Science , Rehovot , Israel

## Abstract

Multi-heme cytochrome c (Cyt*c*) proteins are key for transferring electrons out of cells, to enable intracellular oxidation to proceed in the absence of O_2_. In these proteins most of the hemes are arranged in a linear array suggesting a facile path for electronic conduction. To test this, we studied solvent-free electron transport across two multi-heme Cyt*c*-type proteins: MtrF (deca-heme Cyt*c*) and STC (tetra-heme Cyt*c*). Transport is measured across monolayers of these proteins in a solid state configuration between Au electrodes. Both proteins showed 1000× higher conductance than single heme, or heme-free proteins, but similar conductance to monolayers of conjugated organics. Conductance is found to be temperature-independent (320–80 K), suggesting tunneling as the transport mechanism. This mechanism is consistent with *I*–*V* curves modelling, results of which could be interpreted by having protein-electrode coupling as rate limiting, rather than transport within the proteins.

## Abstract 2

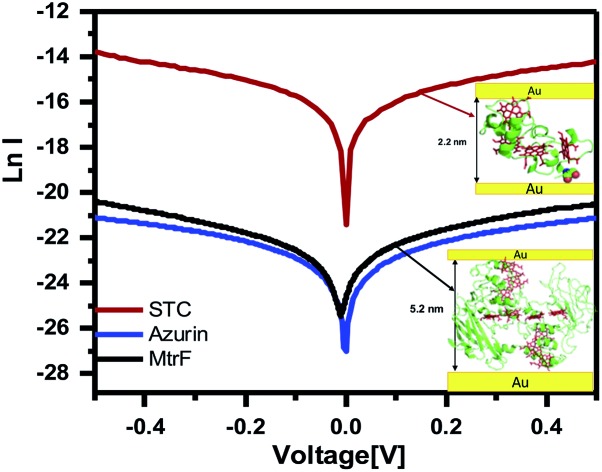
We study solvent-free electron transport across two multi-heme cytochrome c-type proteins, MtrF and STC, and find that they are better at conducting than non- or mono heme proteins.

## Introduction

In extra-cellular respiration an organism oxidizes organic matter inside cells and exports the produced electrons outside the cells, either by communication with other cells^1^ and/or to reduce extracellular oxidized minerals (mostly metal oxides).^2^,^3^ Although the exact mechanism of this electron transport is not resolved, multi-heme *c*-type cytochromes present in cell outer membranes are found to play a central role. Earlier studies found that the heme cofactors of these cytochromes are arranged in a molecular wire-like fashion and that several such proteins span the cellular envelope, allowing electron transfer over long distances (>10 nm).^4^,^5^ Due to these remarkable electron transfer properties, such multi-heme cytochromes are of prime interest for, *e.g.*, potential bioelectronics and bio-sensing. Integrating such proteins into electronic circuits is indeed an exciting prospect.^6^ Hence, it is important to understand the electron transport (ETp) properties of these fascinating proteins on a molecular level. In earlier work it was shown that these cytochromes are essential for efficient electrical transport.^7^

Several mechanisms for electron transfer (ET) in these multi-heme proteins (in solution/in the membrane) have been suggested and analyzed, including band-like transport,^8^ flickering resonance (FR),^9^ superexchange-mediated tunneling (SE),^10^ and charge hopping.^5^ In a recent review we suggested that ET across the fully solvated protein deca-heme Cyt*c* (MtrF) occurs by stepwise (incoherent) transport, electron hopping, between neighboring Fe^2+^/Fe^3+^ heme pairs.^10^ The relatively small electronic coupling between heme cofactors (compared to DNA bases for example) makes FR and SE unlikely as dominant ET mechanisms in multi-heme proteins. Here we address the question of which mechanism(s) dominate in solid state electron transport *via* dry multi-heme proteins, a process which has similarities with, but also clear differences from, ET in aqueous solution, as discussed in detail in ref. 11 and 12.

ETp has been studied in a variety of proteins, using “dry” junctions of monolayers,^11^,^13^,^14^ in which the proteins maintain only structural, tightly bound H_2_O. In such junctions the donor and acceptor, involved in ET in solution,^15^ are replaced by metallic contacts of nm-s to mm size, and electron transport is measured as the current, *I*, as a function of applied voltage, *V* (*I*–*V* characteristics). Such junctions also allow temperature-dependent *I*–*V* measurements.

STM-based solid state *I*–*V* measurements showed deca-heme proteins (MtrF, MtrC and OmcA) to be good electron conductors.^6^,^16^,^17^ As noted in a 2014 summary of literature data,^13^ current values reported by STM measurements have a wide spread, which can be due to factors such as the presence of a vacuum, or air (as in ref. 6, 16 and 17) gap contact geometry, and/or low S/N ratio (relative to larger-area junctions). Also, possible future devices are unlikely to use STM contacts. Thus here we use larger area contacts to help provide insight into multi-heme protein solid state ETp.

To that end we study two multi-heme proteins, the 3-dimensional crystal structures of which have been determined, *viz.* a tetra-heme Cyt*c* protein (STC)^18^ and a larger deca-heme protein, MtrF, one of the largest among the multi-heme cytochromes in extra-cellular electron transport.^19^,^20^

## Results and discussion

**Fig. 1:**
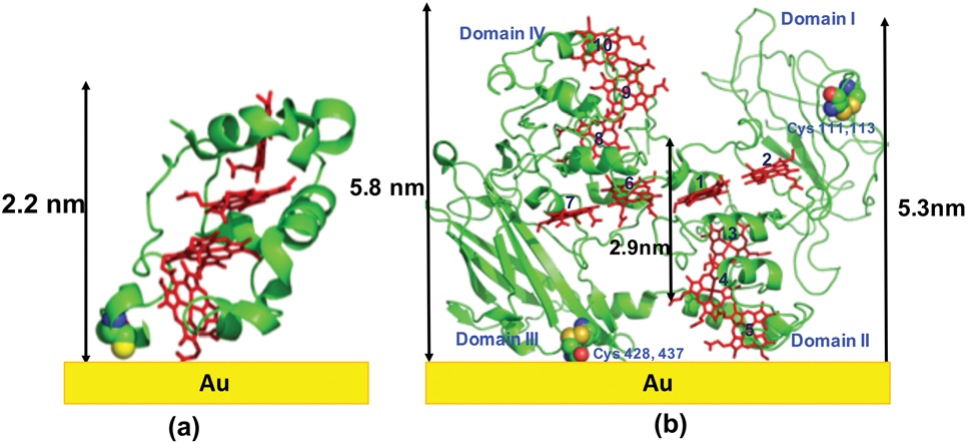
Schematic representation of: (a) STC protein on Au using crystal structure (pdb: 1m1q), (b) MtrF protein on Au using crystal structure (pdb: ; 3pmq). Note, the actual adsorbed structure may differ from the schematic shown here.

We prepared MtrF and STC monolayers between Au electrodes and measured ETp across them. A self-assembled monolayer of MtrF or STC was covalently bonded by a S–Au bond to a polycrystalline Au substrate on one of the relatively exposed cysteine thiolates of these proteins. Since in native STC all eight Cys residues form covalent bonds to heme porphyrins *via* their thiol residues, an additional Cys was introduced by replacing Ser87, which is proximal to Heme IV at the terminus of the approximately linear heme arrangement. In MtrF, cysteines of Domain I (111,115) and Domain III (428,437) are exposed; of these, Domain III cysteines are most likely to form a covalent bond with the Au substrate.^21^ It is possible that when Cys (428/437) forms a Au–S bond with the substrate, heme 5 (Domain II) may also contact the substrate (Fig. 1).

**Fig. 2:**
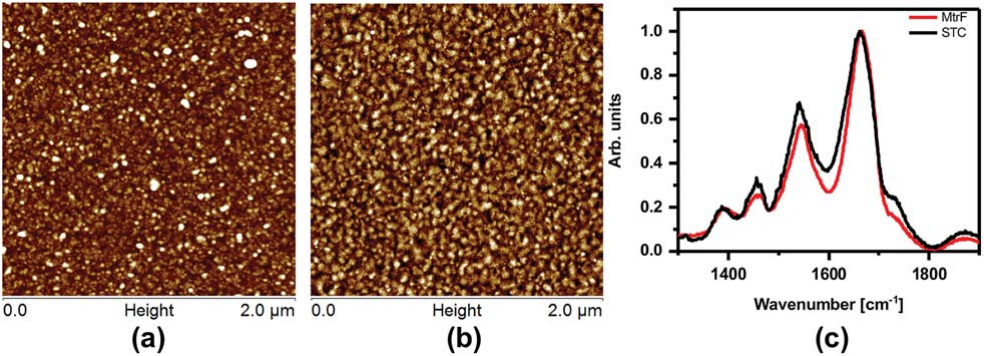
(a) AFM image of MtrF and, (b) STC monolayers on gold using the tapping mode. (c) PMIRRAS of MtrF and STC monolayers on Au.

**Fig. 3:**
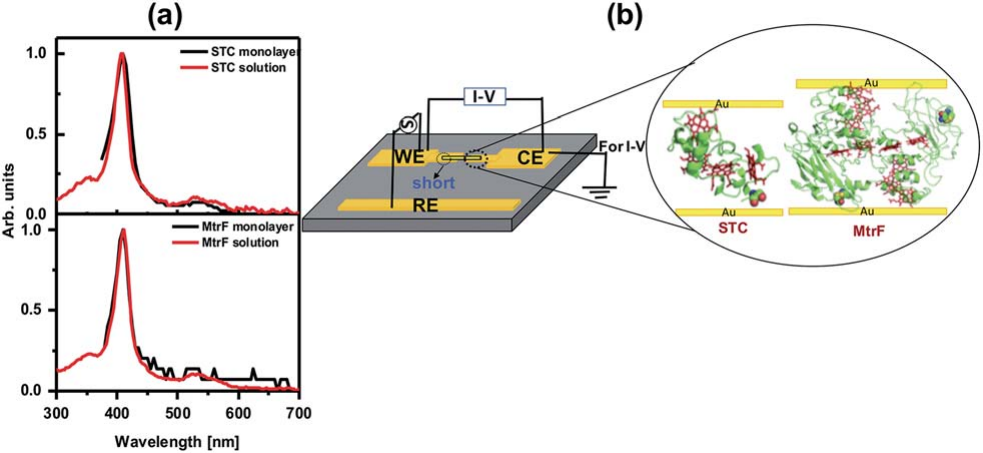
(a) UV-Vis spectra of MtrF and STC proteins in solution and the monolayers on quartz. (b) Schematic of di-electrophoresis, where AC bias is applied between the WE and RE electrode, and the *I*–*V* measurement setup.

The monolayers were formed by incubating the proteins on freshly cleaned and activated Au substrates at 4 °C for 4 h. The resulting protein monolayers were found by ellipsometry to be 4.0 ± 0.1 nm and 2.2 ± 0.1 nm thick, for MtrF and STC, respectively; nano-scratching with the tip of an atomic force microscope (AFM) gave 4.8 ± 0.5 nm and 2.4 ± 0.5 nm for MtrF and STC, respectively (Fig. S3 and S4†). Comparison of these values with the crystal structures of these proteins, indicates that MtrF bound in a roughly upright position; because this protein has a shape, somewhat akin to that of a staggered cross (Fig. 1(b)), we cannot define a unique height, but describe it by three lengths, 5.8 nm and 5.3 nm at the edges and 2.9 nm in the middle, which may be consistent with ∼4.0 nm thickness, in agreement with the ellipsometry-derived value. In STC the theoretical length, determined by crystal structure (pdb ; 1m1q) is 3.7 nm and the observed monolayer width is 2.2 nm (Fig. 1(a)). To fit to the determined thickness, we assume that the protein is tilted from the normal. We note that for both the proteins other orientations are also possible with similar thicknesses. AFM measurements (in tapping mode), indicated that the monolayers were compactly packed with rms roughness of 0.9 nm and 2.1 nm for STC and MtrF, respectively (Fig. 2(a) and (b)). Amide I and amide II peaks at 1664 and 1538 cm^–1^, respectively, in the polarization modulation-infrared reflection-absorption spectra (PMIRRAS), are evidence for the presence of the protein attached to Au (Fig. 2(c)). The integrity (secondary structure) of the protein in the monolayers was confirmed by UV-Vis absorption spectroscopy. To that end monolayers were formed on quartz by S–S linkages using (3-mercaptopropyl)-trimethoxysilane (MPTMS) as the linker. The Soret band of MtrF at 412 nm and STC at 409 nm were found to be the same in the monolayers as in solution (Fig. 3(a)). This confirms that there is no significant change in the molecular environment of the heme groups upon monolayer formation, suggesting that there is no change in the protein conformation.

Creating molecular junctions for *I*–*V* measurements requires care, so as not to damage the protein, *i.e.*, electrical contacts have to be nondestructive. At the same time the junction has to be stable over a wide temperature range to allow low-noise, low-current measurements. To this end, we used two techniques: the first is the “suspended-nanowire” technique,^22^,^23^ with which *I*–*V* can be measured from RT to 80 K (to 10 K, if needed), and the second uses InGa eutectic as the top contact for the RT measurements.^24^ For the suspended nanowire technique, the protein monolayer was coupled covalently, as described above, to pre-patterned Au microelectrodes (Fig. 3(b)). Au nanowires, ∼300 nm in diameter and ∼4 μm long, were trapped di-electrophoretically onto the electrodes (Fig. 3(b)), as reported previously.^25^ In total we made ∼325 Au-protein-Au junctions, of which 65 MtrF junctions and 80 junctions for STC had the desired configuration (nanowire aligned, short on one side, details in ESI† (Fig. 3(b))), based on RT *I*–*V* measurements.

Among these groups of junctions, 10 were chosen that had the statistically most probable currents at 0.5 V, as deduced from a Gaussian fit of currents *via* all junctions, for measurements down to 80 K. Details of statistics of all junctions are given in the ESI (Fig. S1†).

**Fig. 4:**
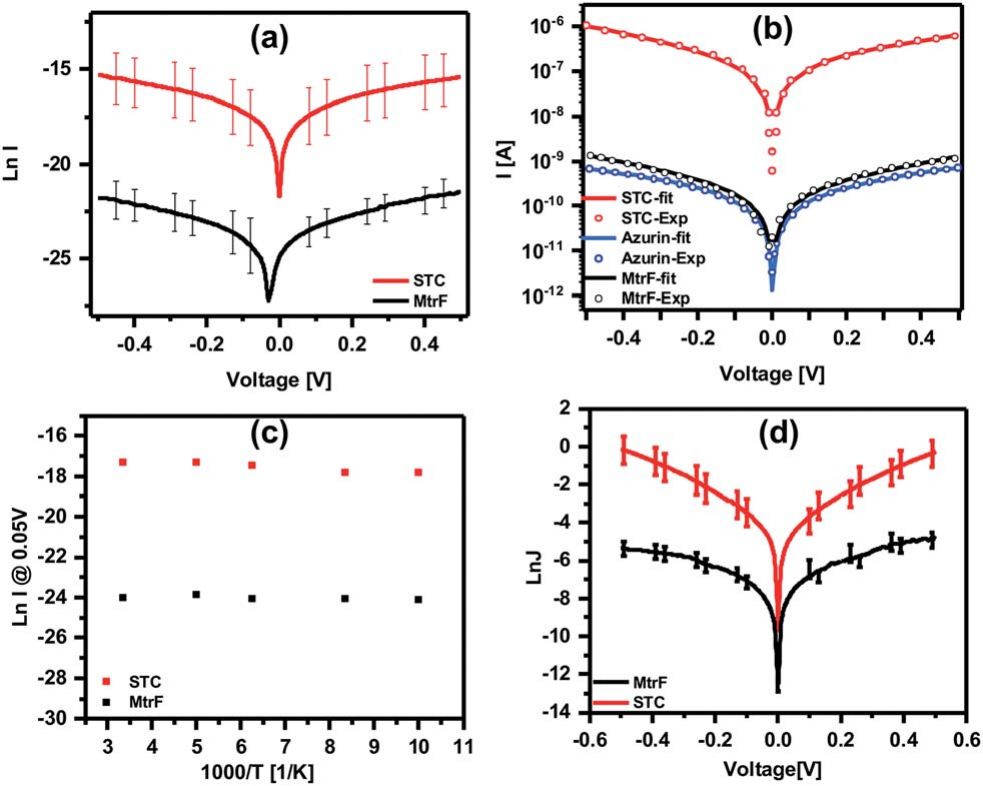
(a) ln *I*–*V* curves obtained using suspended nanowire junctions. (b) Coherent tunneling fit (solid lines) of the experimental *I*–*V* curves (circles) of STC, MtrF and Az (for comparison) (linear *I*–*V* curves are given in Fig. S2†). (c) ln *I vs.* 1000/*T* curves obtained using suspended nanowire junctions; (d) ln *J*–*V* curves obtained using Au substrate with InGa eutectic as the top contact for STC and MtrF monolayers.

Three orders of magnitude higher conductance was observed *via* the STC protein junctions than *via* the blue Cu protein azurin (Az) junctions,^28^ which forms monolayer junctions of similar thickness (2 ± 0.2 nm), (Fig. 4(b)). The comparison measurements on Az were done using the same contacts and measurement method to exclude the effect of contact resistance. For MtrF, even though its monolayer thickness is double that of Az monolayers, *i.e.*, the separation between electrodes is twice that of Az junctions, *I*–*V* curves, very similar to those of Az were observed (Fig. 4(b)). The observed higher conductance of STC compared to that of Az, and the similar conductance of MtrF to that of the much smaller Az, is consistent with the idea that the multi-heme arrays in MtrF and STC can markedly enhance conductance. Similar *I*–*V* curves have been obtained for MtrF and STC when, instead of a Au nanowire, an InGa top contact was employed (Fig. 4(d)). For those experiments monolayers were formed on freshly cleaned Au substrates (100 nm thick) with freshly made InGa as the top contact (scheme given in the ESI†).

To further compare the present results with those obtained with other proteins, current densities for STC and MtrF are estimated assuming the maximum contact area for the nanowire method of 0.03 × 0.1 μm^2^. The current density at 0.05 V was calculated to be ∼0.3 A cm^–2^ and 2 × 10^2^ A cm^–2^ for MtrF and STC, respectively. Earlier we have reported results of ETp measurements *via* monoheme cytochrome c (Cyt*c*) and Az, which impose a similar electrode separation (∼2 nm), and also bind covalently *via* a cysteine thiolate to one of the electrodes. Those proteins, though, were measured in a different device configuration, namely Si/SiO_2_ (1 nm)/linker (0.6 nm)/protein/Hg. The SiO_2_ and (an organic molecule) linker add an insulating layer of ∼16 Å, which lowers the currents by some 5 orders of magnitude, assuming a current decay factor, *β*, across SiO_2_ and alkane chains in solid state junctions (with molecules sandwiched between electrodes) as 0.7 Å^–1^ (for mostly saturated molecules *β* for transport across molecules in these junctions is 0.6–1.0 Å^–1^).^29^–^32^ The current density values after correction (Table 1) for two Cyt*c* mutants (E104C, V11C) that bind to the Au electrode *via* cysteines^26^ and for Az^27^ are 0.2–0.5 A cm^–2^ at 0.05 V. These values are similar to those we have now determined for MtrF (which is double the size of the former proteins; 4 nm). In contrast, for STC, which is the same size (2 nm, namely yielding similar electrode separation as with Az and Cyt*c*) we observe a 10^3^× higher conductance.

**Table 1:** *J*–*V* comparison of literature results reported proteins for in Si/SiO_2_/linker/protein/Hg device configuration

Protein	Thickness [nm]	*J*@0.05 V [A cm^–2^]	Corrected^*a*^ *J* [A cm^–2^]
Cyt*c* (E104C)^26^	2.1	4.9 × 10^–6^	0.49
Az^27^	2.1	1.2 × 10^–6^	0.1
Cyt*c* (V11C)^26^	1.8	2.6 × 10^–6^	0.26

^*a*^For insulating layer of SiO_2_ + linker.

**Fig. 5:**
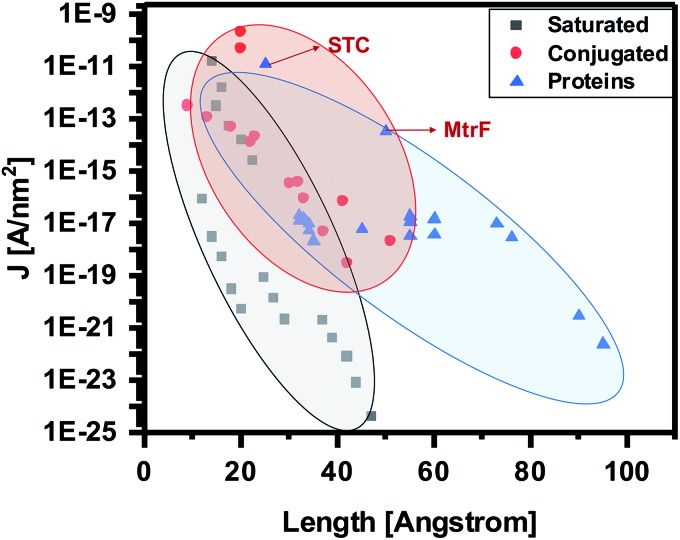
Current densities at 0.1 V [A nm^–2^] as a function of junction width [Å] (the shaded areas provide visual guides only). Extrapolated data points corresponding to MtrF and STC are indicated by arrows. Where applicable, the data are corrected for the current attenuation by 1 nm Si oxide and 0.7 nm saturated organic linker (as described in text). Adapted with permission from ref. 13, Amdursky *et al.*, John Wiley and Sons.

To compare the conductivity of these proteins with other proteins, saturated molecules and conjugated molecules, we update and present here an earlier summary of data (current density (*J* [A nm^–2^]) at 0.1 V *vs.* molecule length (Å) in the junction, (*i.e.*, separation between the electrodes).^13^ The bias of 0.1 V is used, because most data are from the literature and reliable data at lower bias are scarce. By adding STC and MtrF to this plot, we clearly see that their data points are in the region of conjugated molecules (Fig. 5). Thus, at this point we tentatively conclude that monolayers of MtrF and STC conduct like monolayers of conjugated molecules.

Modeling *I*–*V* curves can (help to) identify the current-limiting transport mechanism. In the coherent tunneling model of Simmons the protein matrix is approximated by a single effective energy barrier with height *φ*, length *L* and symmetry factor *α* as fitting parameters,^33^,^34^ *I* ∝ exp[–(*φ* – *αV*)^1/2^*L*].Remarkably, this model yields excellent fits (Fig. 4(b) and S2† solid lines, correlation coefficients = 0.999), but with tunneling lengths (*L* = 1.22, 1.56 and 1.21 nm for STC, MtrF and Az, respectively) that are much shorter than the measured widths of the respective monolayers. For STC and MtrF such lengths are more characteristic of tunneling from an electrode to one of the protein’s hemes. We speculate that subsequent intra-protein conduction, possibly facilitated by heme electronic energy levels, is fast and not resolved in experimental measurements. In this regard, we note that recent calculations predicted that cysteine linkages between the two terminal 1–2 and 3–4 heme pairs of STC would significantly enhance overall electron flow through the solvated protein, due to weak mixing of the S 3p orbital with the Fe-heme d orbitals.^35^ A similar effect may operate for the dry proteins studied here. In solution-phase ET there could also be additional electrostatic effects, due to redox-linked structural modifications, but such effects should be minimal in solid state electron transfer.^36^

To gain further insight into the possible transport mechanisms, temperature-dependence of the ETp *via* the proteins was measured. No temperature dependence of the current at 50 mV was observed from 80 to 300 K (averaged data shown in Fig. 4(c)). Such behavior is consistent with a coherent tunneling mechanism, the model now used to fit the experimental *I*–*V* curves. Since ETp are temperature-independent, the possibility of flickering resonance, as the mechanism for conduction, is unlikely.^10^ Such temperature-independent ETp behavior is also inconsistent with a hopping mechanism.^11^ Theoretically, for electron transfer – a mechanism involving delocalization of orbitals of conjugated molecules^37^ – hopping could be temperature-independent.

It was suggested earlier that super-exchange-mediating tunneling could be the dominant but not exclusive coupling mechanism for long-range ET.^38^ Since the mediating states and energy gaps are rarely identified for this mechanism, it is difficult to define exactly whether it is tunneling or super-exchange-mediated-tunneling. Still tunneling, is the most plausible mechanism as validated by the theoretical fitting of the experimental *I*–*V* curve. This tunneling behavior can be assumed to be intrinsic to the protein, because if transport across the protein monolayers was temperature-dependent, tunneling into and out of the proteins from/to the electrode, would not be sufficient to yield temperature-independent transport.

This can be related to having ET *via* electron tunneling within folded peptide or proteins; occur through covalently linked or hydrogen-bonded pathways between donor and acceptor moieties.^38^,^39^ Thus, at this point we tentatively conclude that MtrF and STC monolayers conduct somewhat like conjugated molecules, *via* tunneling.

## Experimental

### MtrF protein preparation

Cultures of the MR-1 mtr operon mutant (LS623) with the plasmid containing the gene encoding His-tagged MtrF were grown aerobically in Luria–Bertani medium (containing 25 μg ml^–1^ kanamycin) at 303 K overnight. For scale-up, each initial overnight culture (5 ml) was used to inoculate 1 L of fresh Luria–Bertani medium (containing 25 μg ml^–1^ kanamycin). For standard MtrF preparations, 8 × 1 L cultures were grown aerobically at 303 K until the OD_600_ of the culture reaches 0.6 (usually needs 4–5 h). l-(+)-Arabinose was added to reach the final concentration of 1 mM and induced for 17 h. Cells were harvested by centrifugation (6000*g*, 277 K, 15 min), washed and resuspended in 277 K in 50 ml ice cold buffer B (buffer B: buffer A, lysozyme 0.2 mg ml^–1^, DNase 0.01 mg ml^–1^, protease inhibitor and 1% CHAPS [3-[(3-cholamidopropyl) dimethylammonio]-1-propanesulfonate]) (buffer A: 20 mM HEPES [*N*-(2-hydroxyethyl)piperazine-*N*′-ethanesulfonic acid], pH 7.8 and 150 mM NaCl) and kept for stirring overnight at 277 K. Unsolubilized proteins were removed by centrifugation at 12 000 rpm for 30 min. The solubilized protein supernatant was loaded onto 10 ml of Ni^2+^-NTA histidine-tagged agarose column (flow rate of 1.4 ml min^–1^) that has been pre-equilibrated with buffer A in 277 K. The column was washed with 40 ml of each of the following ice-cold buffers in sequential order: buffer D (20 mM HEPES, pH 7.8, 300 mM NaCl, 0.5% CHAPS, 10% glycerol and protease inhibitor), buffer E (buffer D and 10 mM imidazole), and buffer F (buffer D and 40 mM imidazole) in 277 K. The final elution of the protein was obtained with ice cold buffer G (buffer D, 250 mM imidazole and 10% glycerol) and we collected 1.5 ml per fraction at 277 K. The eluted protein was washed and concentrated using 20 mM HEPES, pH 7.8, 30 mM NaCl, 0.17% (wt/vol) CHAPS in 277 K. Aliquots of purified MtrF were stored using 20 mM HEPES, pH 7.5, 100 mM NaCl, 0.5% (wt/vol) CHAPS and 10% glycerol in 193 K. A CD spectrum of the protein was measured to check the protein secondary structure (Fig. S6†).

### STC protein preparation

S87C STC was purified from *Shewanella oneidensis* MR-1 after expression from the corresponding gene was inserted into a pBAD202/D-TOPO vector. An N-terminal Strep II-tag was introduced to facilitate protein purification, full details will be provided elsewhere (van Wonderen *et al.*, DOI: ; 10.1002/cbic.201800313). Protein purity was confirmed by SDS-PAGE, Fig. S7.† LC-MS analysis reveals a single-peak corresponding to a mass of 13 561 Da in excellent agreement with that predicted (13 558 Da) for the mature protein with four covalently bound hemes. Aliquots of purified S87C STC (200 μM) in 20 mM TRIS, 100 mM NaCl, pH 8.5 were stored frozen at 193 K. CD spectrum of the protein was measured to check the protein secondary structure (Fig. S6†).

### Monolayer formation

Au-coated (50 nm) P++ doped Si wafers were cleaned by sonicating for 5 min each in acetone and ethanol, followed by UV/ozone treatment for 15 min. Cleaned Au slides/patterned chips were activated by treatment with hot ethanol for 30 min, and dried with N_2_ and immediately transferred to the protein solution and incubated at 4 °C for 4 h. After 4 h, the slides on which the protein was deposited were gently cleaned with H_2_O and dried with N_2_.

### Ellipsometry

Ellipsometry measurements were performed using a Woollam M-2000 V multiple-wavelength ellipsometer at a 70° angle of incidence. The Cauchy model was used to estimate the protein monolayer thickness.

### AFM imaging

The topography of the self-assembled monolayer of proteins was characterized by AFM in the Scanasyst mode. A Bruker multimode-A and pyrex Nitride probe-Si_3_N_4_ SPM sensor with frequency 67 Hz and force constant 0.32 N m^–1^ were used.

The scratching procedure was performed in contact mode, a 1 × 1 μm^2^ square area was scanned with a large tip force (60 nN). The applied force is sufficiently large to scratch away the monolayer, but not sufficient to scratch the gold surface. After the scratching procedure, we switched back to Scanasyst mode to re-scan over a larger area, centered around the resulting hollow space after scratching (Fig. S1†).

### UV-Vis optical absorption

In solution, these measurements were taken using a nanodrop-2000 spectrophotometer, where the path length is corrected for 1 cm. Protein monolayers were measured using a Quantaurus-QY (absolute PL quantum yield spectrometer), Hamamatsu C11437.

### Circular dichroism (CD)

The CD spectra were measured on a Chirascan spectrometer. The measurements were made using a 1 mm optical-path quartz cuvette. Respective buffers were used as a baseline (Fig. S6†).

### PMIRRAS measurements

PMIRRAS measurements were performed using a Nicolet 6700 FTIR, at an 80° incidence angle, equipped with PEM-90 photoelastic modulator (Hinds Instruments, Hillsboro, OR) with modulation wavelengths of 1600 cm^–1^ for the amide I and II regions. Raw spectra were smoothed and baseline-corrected by a spline algorithm.

### Device fabrication, *I*–*V* measurements and statistics

To fabricate the protein junctions for transport measurements Au electrodes were deposited on a Si wafer by photolithography, yielding a substrate that contains 260 electrode pairs. The proteins were immobilized on the wafer as described above, for monolayer formation. After monolayer formation, gold nanorods were di-electrophoretically trapped to close the circuit, by applying an AC bias between working and reference electrodes, using water as the dielectric medium.^22^,^23^ The final architecture of all measured junctions is similar to the configuration shown in Fig. 4(a), with only a single Au nanorod as a top contact. Since the yield of trapping was only ∼25%, it was rare that two or more Au nanorods bridged two contact pads and this was easily detected by optical microscopy, prior to electronic transport measurements. Next, the samples were loaded on an electrically floating sample stage and were placed in a cryogenic Lakeshore probe station (TTPX). *I*–*V* measurements were performed to assess the transport efficiency across peptide monolayers, using a Keithley 6430 Sub-Femto amp Source-Meter, with a voltage scan rate of 20 mV s^–1^ in a vacuum of 10^–5^ mbar. For all measurements, a specific side of the junction was grounded, while the other one was biased, in a consistent manner (in order to ensure that the bias polarity was in the same direction for all measurements). In each set of experiments, scans were acquired that started and ended at 0 V (*i.e.*, voltage sweep was 0 → –0.5 V, –0.5 V→ 0.5 V, 0.5 V → 0 V), to check if features in the *I*–*V* behavior originate from the polarity of the initial voltage that is applied and from the scan direction (hysteresis check). All the aligned nanowire junctions ∼25% of 1300 junctions (5 chips with 260 junctions/chip) were measured: ∼40% of the remaining 325 junctions showed no currents (possibly these were double junctions, rather than the desired single junction, with one nanowire/substrate contact shorted); ∼35% (98) junctions were short circuited; and ∼25% (∼80) of the junctions showed single junctions with currents which fit that. The most probable current range for the protein was determined by statistics, for all currents, frequency of occurrence at 0.5 V is checked and frequency count histogram was fitted to Gaussian, from the FWHM, the desired range of current is calculated (Fig. S2†). In case of MTRF, current range at 0.5 V was found to be 0.1–5 nA and for STC it was found to be 0.01 μA to 5 μA. To measure down to 80 K, we have chosen 10 junctions each with current values at peak maxima, for MTRF 0.5–1 nA, for STC 0.1–0.5 μA.

### Coherent tunneling model

The experimental *I*–*V* data shown in Fig. 4(b) (main text) and S2† were fit to the following tunneling expression.^33^,^34^

1 *I*(*V*) = *Ai*(*V*)

2
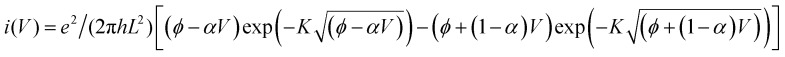

3
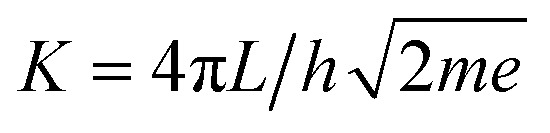
where *I* is the current, *i* the current density, *A* the contact area, *e* the elementary charge, *h* Planck’s constant and *m* the electron mass. The fit parameters are the barrier height *φ*, barrier length *L*, and symmetry factor *α*. The contact area was obtained from the measured current and the estimated current density at 0.05 V (see main text), *A* = *I*(0.05 V)/*i*(0.05 V), *I*(0.05 V) = 6.0 × 10^–8^ A and 9.9 × 10^–11^ A for STC and MtrF, respectively, and *i*(0.05 V) = 200 A cm^–2^ and 0.3 A cm^–2^ for STC and MtrF, respectively. Numerical values of the fit parameters are summarized in Table S1.†

## Conclusion

We conclude that the multi-heme proteins, MtrF and STC are significantly better at conducting than non- or mono-heme proteins. These multi-heme proteins conduct, somewhat like conjugated organic molecules in the dry phase. The electron transport process, being temperature independent and examined for coherent tunneling fit with the experimental *I*–*V* results, reveals that the transport is indeed by tunneling mechanism. Whether coherent tunneling takes place over the full electrode-electrode distance, or over a smaller distance from one electrode to the nearest heme followed by fast intraprotein conduction not resolved in the experimental measurements, and is still open for debate. The relatively short tunneling distances obtained from the Simmons model would support the latter interpretation. As we have no indication of any resolvable structural changes in the proteins, we assume that the electron migration rate within the proteins, whether by ET or ETp, is comparable. If so, then these results present a significant challenge to our current understanding of electron transfer and transport *via* proteins, and as such may stimulate re-evaluation of existing models. In addition, these results follow other indications that solid-state conduction across proteins is limited by the coupling to the electrodes.

## Conflicts of interest

There are no conflicts to declare.

## Supplementary Material

Supplementary information
