# Supplementary material for: Direct evidence for heme-assisted solid-state electronic conduction in multi-heme c-type cytochromes
Source: Chem Sci. 2018 Jul 27;9(37):7304–10. doi: 10.1039/c8sc01716f (PMC6166575; doi:10.1039/c8sc01716f)
Supplement: Supplementary file 1 [file SC-009-C8SC01716F-s001.pdf]

Electronic Supplementary Material (ESI) for Chemical Science.
This journal is © The Royal Society of Chemistry 2018

Supplementary Material

Direct evidence for heme-assisted solid-state electronic conduction in multi-heme *c*-type cytochromes

Kavita Garg,[a] Mihir Ghosh,[a] Tamar Eliash,[a] Jessica H. van Wonderen,[b] Julea N. Butt,[b] Liang Shi,[c] Xiuyun Jiang,[d] Futera Zdenek,[d] Jochen Blumberger,[d] Israel Pecht,[e] Mordechai Sheves,[a*] David Cahen.[e*]

[a] Weizmann Institute of Science, Rehovot, Israel. [b] School of Chemistry, School of Biological Sciences, University of East Anglia, Norwich Research Park, Norwich, NR47TJ, UK. [c] Department of Biological Sciences and Technology, School of Environmental Sciences, China University of Geosciences, Wuhan, China 430074. [d] University College London, Department of Physics and Astronomy, Gower Street, London WC1E 6BT, UK.

Supplementary figures:

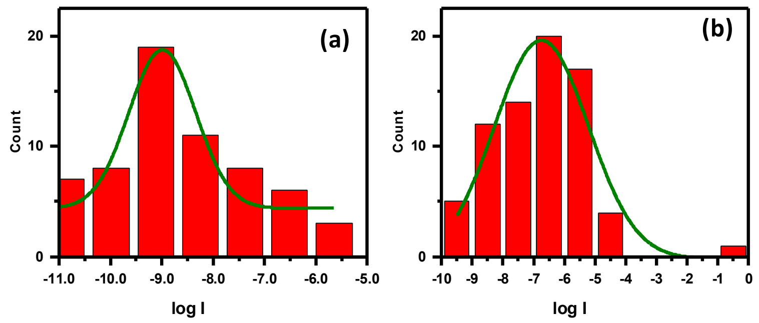

Figure S1. *Statistics of I-V measurements by nanowire method at 0.5V; (a) MtrF (b) STC.*

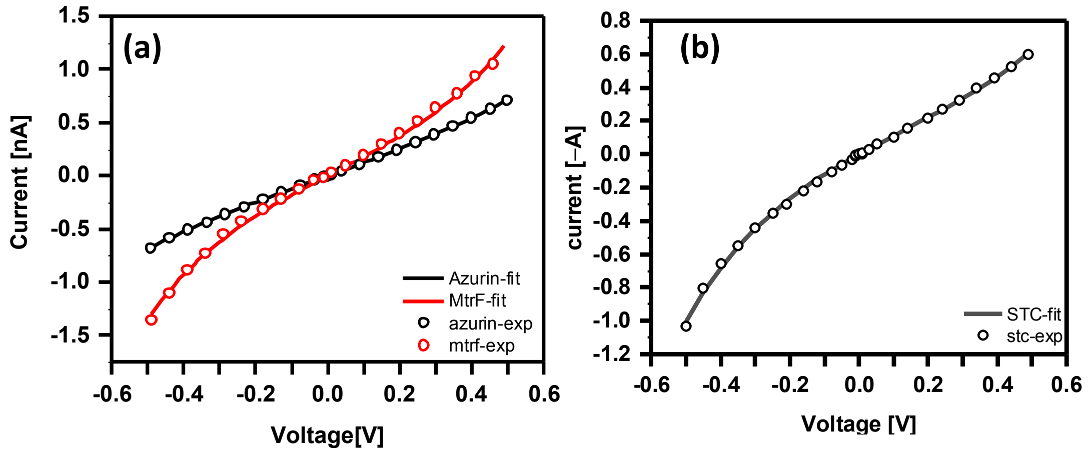

Figure S2. *Coherent tunneling current fitting of experimental of I-V data by nanowire method; (a) MtrF and azurin (b) STC.*

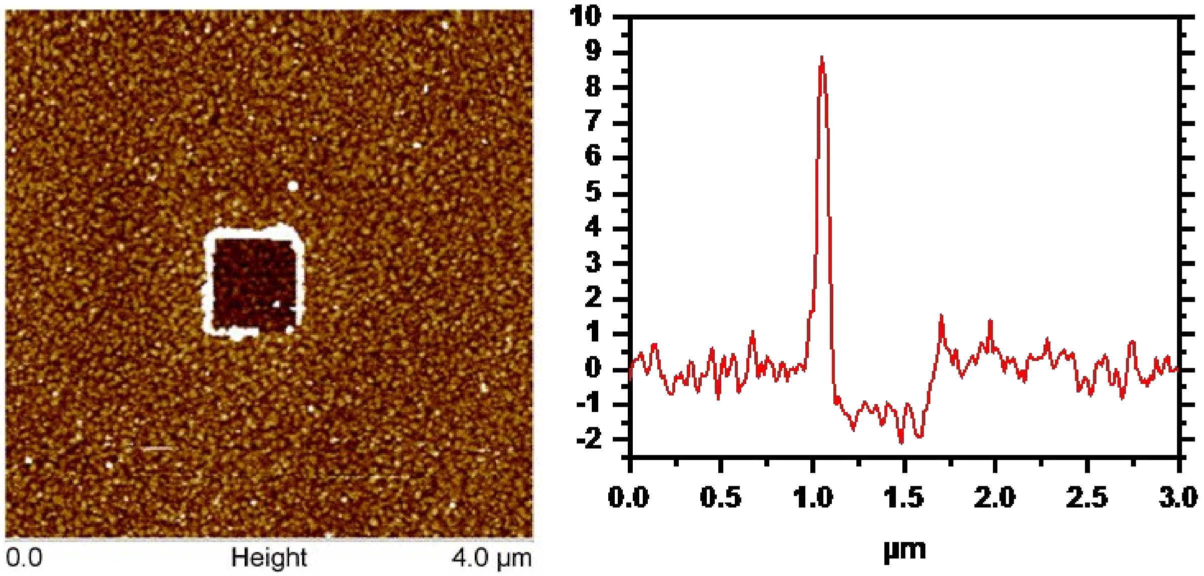

Figure S3. *AFM scratching of STC monolayers*

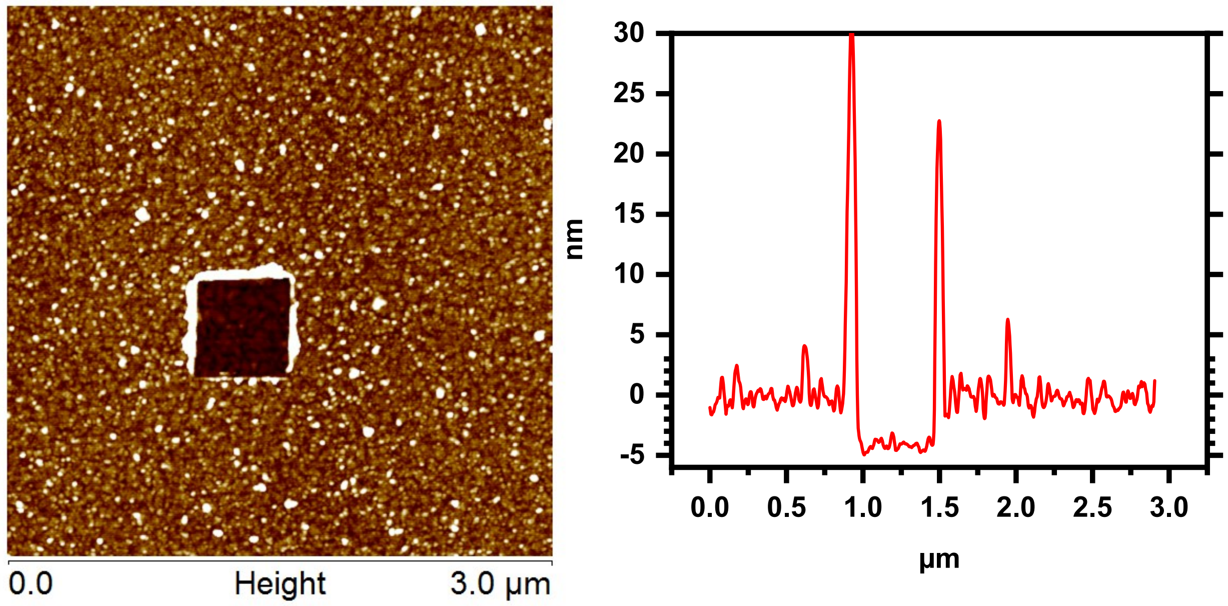

Figure S4. *AFM scratching of MtrF monolayers*

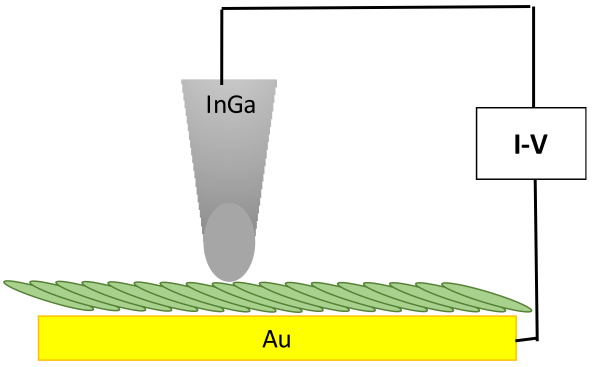

Figure S5. *I-V measurement setup for InGa top contact*

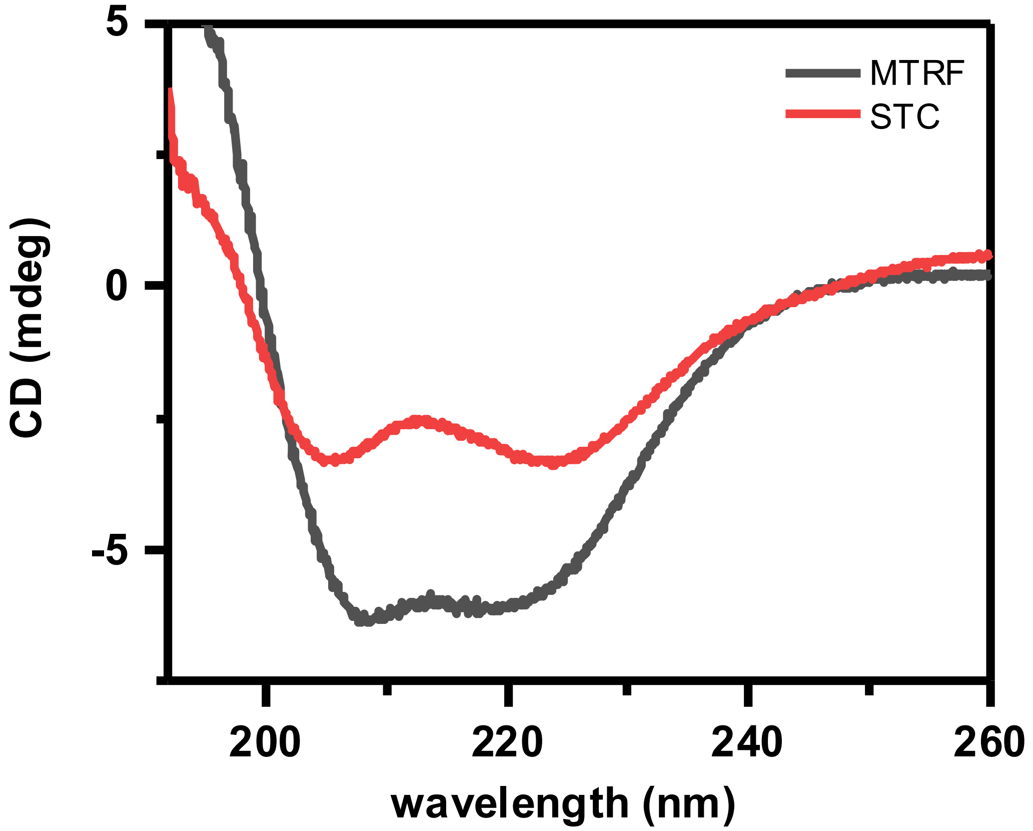

Figure S6. *CD of MtrF and of STC in solution.*

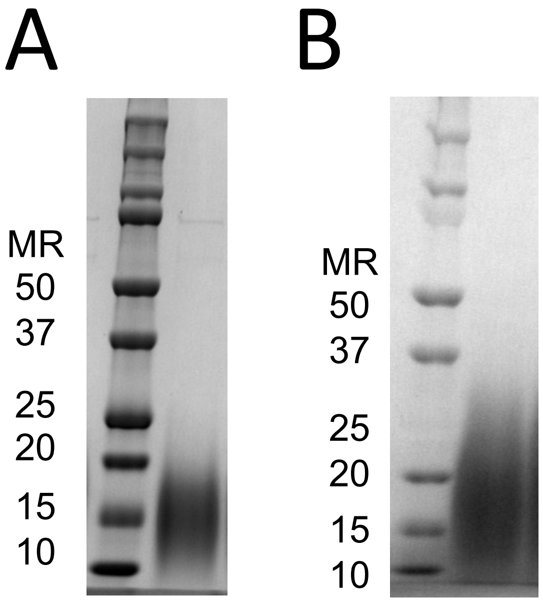

Figure S7. *SDS-PAGE gels for purified S87C STC with molecular weight markers (kDa). To obtain monomeric forms, 1 mM TCEP was added to S87C STC prior to loading. A] Coomassie stained, B] Heme stained.*

Table S1: Summary of fit parameters for coherent tunneling model, Eqs. S1-S3.

	STC	MtrF	Azurin
L (nm)	1.22	1.56	1.21
Φ (eV)	1.13	1.53	2.77
α	0.42	0.49	0.51
corr[a]	0.999	0.999	0.9999

[a]correlation coefficient.
